# Linguistic and Cognitive Skills in Sardinian–Italian Bilingual Children

**DOI:** 10.3389/fpsyg.2015.01898

**Published:** 2015-12-17

**Authors:** Maria Garraffa, Madeleine Beveridge, Antonella Sorace

**Affiliations:** ^1^Department of Psychology, School of Life Science, Heriot-Watt UniversityEdinburgh, UK; ^2^School of Philosophy, Psychology and Language Sciences, The University of EdinburghEdinburgh, UK

**Keywords:** minority languages, grammar, bilingualism, executive functions, Sardinian, object relatives

## Abstract

We report the results of a study which tested receptive Italian grammatical competence and general cognitive abilities in bilingual Italian–Sardinian children and age-matched monolingual Italian children attending the first and second year of primary school in the Nuoro province of Sardinia, where Sardinian is still widely spoken. The results show that across age groups the performance of Sardinian–Italian bilingual children is in most cases indistinguishable from that of monolingual Italian children, in terms of both Italian language skills and general cognitive abilities. However, where there are differences, these emerge gradually over time and are mostly in favor of bilingual children.

## Introduction

Multilingualism is the norm in many parts of the world: according to some conservative estimates (Tucker, 1998), at least half of the world’s population speaks two or more languages. While many factors contribute to the increase in bilingualism in Europe, including transnational population mobility and the status of English as a *lingua franca*, bilingualism in regional minority languages is declining due to the lack of intergenerational transmission (see Romaine, 2007; Extra and Gorter, 2008). Fewer parents speak minority languages to their children because of their perceived lack of ‘usefulness’ and other more general misconceptions on early bilingualism. A similar gap is seen in research into different types of bilingualism. Bilingualism is the object of much linguistic and cognitive research that investigates different aspects of development and use, but bilingualism involving minority languages has not received the same attention as bilingualism involving prestigious languages with wide currency. This paper makes a contribution to redressing the balance by presenting the results of a pilot study on the linguistic and cognitive abilities of children who speak Sardinian as a minority language and Italian as the majority language. We will first briefly summarize research on language development in bilingualism, with an emphasis on grammatical models and general cognition. This will be followed by some notes on the status of Sardinian as a minority language. We will then present the methods employed in the collection of data and the results of statistical analyses. Finally, the data will be discussed against the wider context of bilingualism in regional minority languages.

### Language and Cognition in Bilingual Children: Highlights of Previous Research

#### Morphosyntactic Development

The central question underlying research on bilingual syntactic acquisition is whether bilingual children differentiate their two languages at all stages of development, and whether the two language grammars influence each other. In spite of consensus in early research that bilingual first language acquisition is characterized by independent and parallel acquisition of syntax (Meisel, 1989; De Houwer, 1990; Genesee et al., 1995), more recent research has revealed a more nuanced picture.

For example, Dopke (1998) and Yip and Matthews (2007) reported cross-linguistic effects of one language on the other at the syntactic level, from the dominant language, or the language of the environment, to the weaker language. The effects of dominance and of the amount of input in the weaker language are solidly attested. Bernardini and Schlyter (2004) found syntactic effects of Swedish on Italian and French in Swedish-dominant bilinguals; Meisel (2007) found a lower mean length of utterance (MLU) but no divergent syntactic patterns in the weaker French of French–German bilinguals; Gathercole (2007) reported that monolingual English children outperform school-age English–Spanish bilinguals who are dominant in Spanish in measures of both mass/count distinction and gender; Paradis et al. (2011) studied regular and irregular English past tense in English-dominant and French-dominant children, reporting that English-dominant children scored lower than monolinguals only for irregular forms, but French-dominant children scored lower on both English regular and irregular forms. A similar but more qualified conclusion was reached by Blom (2010) who showed clear input effects in younger Dutch–Turkish bilinguals: Turkish-dominant children were delayed in acquiring the relationship between finiteness and subject realization in Dutch, but Dutch-dominant children were not. Blom argued that reduced input quantity does slow down grammatical development. However, these differences are limited to the weaker language of bilingual children, and are visible only in situations of clearly reduced input. When bilingual children receive balanced input in the two languages, other factors such as age of first exposure and consistent input for a particular structure play an important role. Unsworth et al. (2014), for example, showed that highly regular and consistent grammatical gender in Greek is acquired in similar ways by simultaneous English–Greek bilinguals and monolingual Greeks, but the similarity breaks down in consecutive older bilingual children. In contrast, the inconsistent system of gender in Dutch is acquired late both by monolingual Dutch children and by English–Dutch bilinguals, regardless of age of first exposure.

Cross-linguistic effects in bilingual development may be selective and asymmetric for other reasons. Müller and Hulk’s (2001) seminal work argued that structures at the interface between morphosyntax and discourse are vulnerable to cross-linguistic influence in early bilingual language development, but core syntactic structures are not. Subsequent research refined this hypothesis. For one thing, it was shown that not all structures that satisfy the ‘interface’ requirements show evidence of cross-linguistic influence (see Unsworth, 2005 on optional infinitives in English–German bilinguals). Furthermore, phenomena at the syntax-pragmatics interface, such as the interpretation of pronominal anaphoric forms, take longer to be acquired than phenomena at the syntax-semantics interface such as the use of determiners in generic vs. specific plural nouns (Paradis and Navarro, 2003; Serratrice et al., 2004; Serratrice, 2007; Sorace and Serratrice, 2009). An emerging striking generalization is that delays and inconsistency at the syntax-pragmatics interface have been attested in bilingual children regardless of whether the two languages are grammatically similar, and have been found to also characterize late bilinguals in both the L2 and the L1 (Sorace, 2011, 2012). These parallelisms suggest that the reason for the generality of these effects in bilingualism may lie in extra-linguistic general cognitive factors, rather than in language-specific effects of one grammar over the other.

In the study reported in this paper we investigate possible effects of Sardinian on Italian in school-age children who grow up in an environment where Italian is the majority language, but who are exposed to proportionally more Sardinian in early childhood until they start schooling. We chose to focus on comprehension of a range of productive syntactic structures of Italian with different degrees of complexity, as a first step toward establishing whether there are indeed effects of Sardinian on Italian at the beginning of the schooling process and whether these effects decrease with more exposure to Italian. The structures tested were active and passive structures, coordination, dative structures, topicalisation/left dislocation, and subject and object relatives (see **Tables 1** and **2** and see Studies of Regional Minority Languages).

**Table 1:** Sentence structure types tested in the COMPRENDO receptive test of Italian and translations in Sardinian.

Sentence type	Italian example	Sardinian translation
Active	Il cane morde il gatto *The dog bites the cat*	Su cane mossigat (a) sa gato *The dog bites the cat*
Dative	La mamma dà la torta al bambino *The mother gives the cake to the boy*	Sa mamma li dat su durce a su pitzinneddu *The mother to-him gives the cake to the boy*
Coordinate object	Il bambino insegue il cane e il gatto *The boy chases the dog and the cat*	Su pitzinneddu pressighit su cane e sa gato *The boy chases the dog and the cat*
Passive	Il bambino viene inseguito dal cane *The boy is chased by the dog*	Su pitzinneddu est pressighidu dae su cane The boy is chased by the dog Su pitzinneddu lu pressighit su cane *The boy him chases the dog*
Topicalised OSV-number mismatch	La bambina, i nonni la inseguono *The girl, the grandparents chase her*	Sa pitzinnedda la pressighint sos mannois* The girl, the grandparents chase*
Subject relative	Il nonno spinge il cane che morde il gatto *The grandfather pushes the dog that bites the cat*	Su mannoi ispinghet su cane chi mossigat sa gato *The grandfather pushes the dog that bites the cat*
Object relative	La mamma guarda il cane che il bambino insegue *The mother looks at the dog that the boy chases*	Sa mamma abbaidat su cane chi su pitzinneddu pressighit *The mother looks at the dog that the boy chases*

**Table 2:** Mean age, cumulative length of exposure to Italian, and performance on background tests: RAVEN, PPVT-4, digit span, and non-word repetition tasks across groups (raw scores and SD).

Group *N* = 85	Age Years Mean (*SD*)	UBILEC Cumulative exposure index	RAVEN Mean (*SD*)	PPVT- 4 Mean (*SD*)	Digit span Mean (*SD*)	Non-word repetition Mean (*SD*)
Bilingual Y1 *N* = 18	6.65 (0.32)	1.0	24.00 (3.56)	96.83 (7.96)	4.40 (0.60)	13.45 (2.19)
Bilingual Y2 *N* = 22	7.80 (0.47)	0.8	24.41 (3.9)	102.50 (13.29)	5.10 (0.70)	11.75 (1.48)
Monolingual Y1 *N* = 20	6.60 (0.31)	4.4	22.80 (3.21)	99.35 (16.11)	4.80 (0.68)	12.95 (2.29)
Monolingual Y2 *N* = 25	7.68 (0.26)	4.6	24.68 (3.74)	100.24 (11.53)	4.84 (0.61)	12.48 (2.06)

#### Cognitive Effects of Bilingualism

Recent research on bilingualism has revealed that the bilingual experience can have effects on general cognition beyond the language domain (see Bialystok, 2009; Baum and Titone, 2014; Costa and Sebastian-Galles, 2014 for overviews). The most consistent empirical finding is that of advantage in attentional aspects of executive functions. Adopting Miyake and Friedman’s (2012) tripartite distinction of executive functions into updating, shifting, and inhibition, one can say that the jury is still out as to precisely which component(s) are affected by bilingualism. What seems to be clear, however, is that some of these effects are greater in bilingual children and older bilingual speakers than in young bilingual adults, possibly because the effects are more visible when executive functions are either developing or declining but are not at their peak (Craik and Bialystok, 2006). In bilingual children, advantages have been found in metalinguistic tasks requiring a focus on form in the presence of a distracting meaning (Bialystok, 1988, 1992). Executive control may be involved in these tasks in order to ignore the meaning and focus on form. Similarly, advantages have been reported for the development of theory of mind (ToM) and pragmatic/conversational abilities (Goetz, 2003; Siegal et al., 2009, 2010), which may involve executive control in the suppression of ones’ own perspective when focusing on that of others.

Discussions of the reasons behind the bilingual advantage rely on defining how the two languages are processed in the brain, how they are accessed and how they interact with one another. One theory that has attracted much consensus is the joint activation model (Green, 1998), which assumes that both languages are always active regardless of whether the context of communication is monolingual or bilingual. The bilingual speaker therefore has to suppress the language not in use, or alternatively to enhance activation of the target language (Costa et al., 2006). The core of the debate revolves around whether the main advantage displayed by bilinguals is the ability to focus on the desired information while ‘ignoring’ (but not ‘inhibiting’) the distracting information, or whether it crucially lies instead with the ability to *inhibit* irrelevant information or distracters (Bialystok, 2009). While Bialystok (2009) puts more weight on inhibitory control as the key force in the language selection process, she recognizes that one mechanism is not necessarily mutually exclusive of the other: it could be the case that both inhibiting and ignoring can allow the bilingual speaker to use one language without interference from the other (see also Adaptive Control Hypothesis; Green and Abutalebi, 2013). Depending on the type of bilingual experience and how these experiences ‘sculpt’ the bilingual brain, one might expect to see different effects on general cognitive abilities. Bilinguals have been shown in some studies to outperform monolinguals not only in trials that require inhibitory control of distracting information, but also in trials where no distracting information is present: this fact suggests that the cognitive abilities affected by bilingualism may be broader and more general than inhibitory control (Hilchey and Klein, 2011). It should be added that a bilingual advantage has also been found in a few studies of infants (see, e.g., Kovács and Mehler, 2009) who do not yet experience language control in production (but see Blumenfeld and Marian, 2011, on how inhibitory control affects comprehension too).

It is possible that different types of bilingual experience may lead to different (or null) effects on cognitive abilities. For instance, Costa et al. (2009) proposed that speakers with highly separated and predictable domains of use for each language – thus with a low level of switching required – may not show advantages. Similarly, Prior and Gollan (2011) suggest that an advantage in task switching may arise only in bilinguals who frequently switch between languages. The presence of bilingualism in all societal contexts may have an effect, as well as the relatedness of language pairs (Costa et al., 2009; see Grohmann, 2014 on ‘language proximity’ as an important factor for simultaneous child bilingualism). With this in mind, it is important to gather data from different types of bilinguals, with different language backgrounds, to gain a fuller picture of the effects of bilingualism in particular domains.

The most recent debate has centered in particular on the replicability of the ‘bilingual advantage,’ which a number of studies have failed to find (Paap and Greenberg, 2013; Duñabeitia et al., 2014; Paap, 2014). Some researchers interpret these null results as questioning the validity of previous results showing a bilingual advantage (see de Bruin et al., 2015; Valian, 2015). Others view the failure to replicate in some studies as a normal manifestation of variation due to interactions with poorly understood factors (age at testing, language combination, patterns of bilingual language use, education levels, societal attitudes, etc.), and ultimately as a welcome incentive to carry out more research in different bilingual settings. Bilingualism with regional minority languages, in particular, is a setting that has generated a sparse and inconsistent picture (see below). Furthermore, there is a need for more research that compares child and adult bilinguals in order to trace the developmental trajectory of the effects of bilingualism over the lifespan. More research is also needed to compare children who become bilingual at different stages of childhood (see Bialystok et al., 2012). The Sardinian context offers a unique opportunity to study the emergence of bilingualism in a minority language and its effects over time in school-age children who receive instruction in the majority language.

### Bilingualism in Regional Minority Languages

As a broad group, minority languages tend to differ in significant ways from majority languages with respect to (i) quality and quantity of input, (ii) social status and attitudes toward the language, and (iii) motivation toward bilingualism. First, a significant proportion of languages of the world today are currently facing a drastic decline in numbers of speakers (Nettle, 1999; Crystal, 2000; Grenoble and Whaley, 2006). Thus, the range of different speakers a child acquiring the language has exposure to may be limited. Having exposure to a range of different speakers is important in the acquisition of any language and may affect the child’s language proficiency (Houston and Jusczyk, 2000). It can also be the case with minority languages (likely more so than with majority languages) that teachers, parents and others passing on the language to the child may be second language speakers/learners themselves. This situation inevitably generates a different type of exposure for the child learning a minority language, compared with a child learning a majority language and who is likely to have input from a wide range of different, native speakers. Second, the often unstable or turbulent political history of the minority language may negatively affect the linguistic experience of children. This may be manifested, for example, in the form of lack of institutional support toward the language or in parental lack of motivation to speak the language due to its perceived inutility (Crystal, 2000). Sardinian is no exception in this broad picture.

#### Studies of Regional Minority Languages

The cognitive effects of bilingualism in minority languages have been investigated in a limited number of studies, which provide an inconsistent picture. On the one hand, no bilingual advantage in executive functions was found in studies of Welsh–English bilinguals (Gathercole et al., 2014) and Basque–Spanish bilinguals (Duñabeitia et al., 2014). These studies focused on communities where the minority language has an officially recognized and protected status, yet no differences were reported. On the other hand, other studies do show an advantage for bilingual speakers of minority languages. Antoniou et al. (2014) tested children in Cyprus who were bilingual (or ‘bilectal’) in Greek and Cypriot Greek, and found that they outperformed age-matched monolingual children on all measures of cognitive control, although not on all vocabulary measures. Lauchlan et al. (2013) compared Gaelic–English and Sardinian–Italian bilingual and monolingual English and Italian children in Scotland and Sardinia on measures of cognitive control, problem-solving ability, metalinguistic awareness, and working memory. The results showed a global bilingual advantage over the monolinguals in two of the four measures used. In addition, the bilingual Scottish children significantly outperformed the bilingual Sardinian children: this difference is interpreted as a consequence of the fact that the bilingual Scottish children received Gaelic-medium education, in contrast to the Sardinian bilingual children who mostly speak the minority language only at home. Finally, Vangsnes et al. (2015) looked at the effects of bidialectal literacy in the two Norwegian standards Nynorsk (the minority system) and Bokmål (the majority system) in the minority group of pupils who are schooled in Nynorsk. The data show that these pupils perform better than average in national tests of English, reading and arithmetic once socio-economic factors are controlled for.

#### Sardinian

Most scholars regard Sardinian as a separate Romance language (Harris and Vincent, 1988; Posner, 1996). The long period of independent development following the fall of the Roman Empire distinguishes it clearly from other Romance languages, and it is not intelligible to speakers of Italian. However, the present-day sociolinguistic reality is such that most speakers of Standard Italian probably consider it to be a “dialect” of Italian. Sardinian tends to be used in local and/or informal settings, while Standard Italian is the expected language in official contexts, in cities, in church and in school.

The Sardinian regional government commissioned a comprehensive study of language use in the early part of the 21st century (Oppo, 2007), based on a sample of approximately 2400 respondents aged 15 and above from all over the island. According to this study, nearly 70% of respondents reported that they speak a “local language” (term referring to any local variety of Sardinian, as well as to the other languages spoken by small communities on the island such as Gallurese and Catalan) and nearly 30% said they understood one but did not speak it; only 2.7% claimed no knowledge of a local language. The study also confirmed that there are substantially fewer speakers of local languages in towns and cities with more than 20,000 inhabitants than in villages and rural areas. There are probably no monolingual speakers of Sardinian anywhere on the island, though there are certainly elderly speakers who are more at ease in Sardinian than in Italian.

Oppo’s study also briefly reports the results of a similar survey of approximately 270 children under 14. The proportions are markedly different from the adult figures: just over 40% reported speaking a local language; just over 35% said they understood but did not speak a local language; and more than 20% said they neither spoke nor understood a local language. The substantially smaller proportion of children than adults who report using a local language clearly points to the endangered status of Sardinian as a whole. There are still parts of the island, such as the Nuoro province in central Sardinia, where children routinely learn Sardinian in the family before learning Italian at school, but there are many more children who learn Italian in the family and never acquire Sardinian.

#### Sardinian and Italian: A Brief Comparison

Although the grammars of Sardinian and Italian share a common origin, they are not identical – for a general description of the syntactic differences between the two languages, see Jones (1993) and Bolognesi (2013). One difference that is relevant for the structures in focus here concerns the passive structure, for which dialectal variation is observed. In particular, the passive is possible but dispreferred by speakers in the central Sardinian areas where the data were collected, whereas speakers from southern regions find it more acceptable, possibly because of the stronger influence of Italian. Other relevant differences are the prepositional marking of direct objects and the clitics doubling with indirect objects, which are common in all varieties of Sardinian but ungrammatical in Italian.

Another point of interest is how bilingual children deal with structures that have been reported to be developmentally late in monolingual acquisition. A well-known example is relative clauses, which have been identified in several studies as difficult to acquire in different languages (see Adani, 2011 for an overview). Object relatives, in particular, develop rather late in monolinguals. A theoretical account of the source of complexity for object relatives originally proposed for adults with acquired language disorders and children with atypical language development (Garraffa and Grillo, 2008; Grillo, 2008; Contemori and Garraffa, 2010) and successfully extended to typical language development (Friedmann et al., 2009), is in terms of the intervention of the lexical subject on the long distance dependency established between the relative head and its original position. This intervention effect is schematically shown below.

**Figure:**
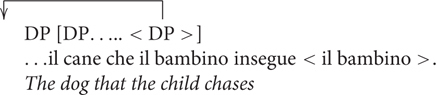


Object headed relative clauses are more difficult to produce and comprehend compared to subject headed relative clauses. Production studies in fact reveal different strategies adopted by monolingual speakers in order to produce simpler sentences not subject to intervention in place of an object relative, but still preserving the meaning of the sentence (Contemori and Belletti, 2012).

One well-attested strategy to avoid intervention is replacing object relative with a passive object relative, POR (i.e., *Il cane che è inseguito dal bambino*, ‘the dog that is chased by the child,’ in place of *il cane che il bambino insegue*, the dog that the child chases). In order to use the POR strategy productively it is necessary to fully master the passive morphology that is the trigger for the movement of the verb phrase not subject to intervention (see Collins, 2005 for a detailed approach on passives sentences). Another productive strategy to avoid the complexity of the object relative was reported by Adani et al. (2010), where an ameliorative effect on comprehension of ORs was attested in the case of sentence with argument number mismatch (i.e., *Il leone che I coccodrilli stanno toccando è seduto per terra* ‘the lion-SG that the crocs-PL are touching is sitting-SG on the floor’). Both the passive structure and verbal inflection strategies required a full command of the morphosyntactic aspects of the language. Adults as well as monolingual children at young ages either did not produce object relatives, replacing them with passive object relatives, or are more likely to produce object relatives when there is a morphological mismatch between the arguments. The question is whether these difficulties would affect bilingual children to the same extent as monolinguals in a comprehension task, given that Sardinian relative clauses are structurally similar to Italian relative clauses (see **Table 1** below).

#### Research Questions

This pilot study aims to address these questions:

(a) Do Sardinian–Italian bilingual children have a disadvantage compared with monolingual Italian children in their comprehension abilities of Italian when they start being schooled in Italian? If they do, is the disadvantage manifested only for particular structures? If there is a difference between bilingual and monolingual children, does it change over time due to age and more experience of Italian in the school setting?
(b) Do Sardinian–Italian bilingual children have an advantage compared to monolingual Italian children in general cognitive abilities related to attentional control and executive functions? If there is a difference between bilingual and monolingual children, does it change over time due to age and more experience of Italian in the school setting?

## Materials and Methods

### Participants

Ninety five children from nine primary schools in the towns of Fonni, Orgosolo, Mamoiada, Nuoro, Desulo, Tonara, Bitti, Lula, and Orune, all in the Nuoro Province, participated in the study. All children were attending the first or the second year of primary school, where the language of instruction is Italian. 10 children were excluded because they did not meet standardized criteria in one or more screening background tests (see below). The final sample included 85 children whose ages ranged from 6 to 9 years and 1 month. For the majority of bilingual children, exposure to Italian occurred at school; therefore, the amount of time spent in education was considered an important predictor of Italian competence. At the time of testing, 18 of the bilingual children and 20 of the monolingual children were finishing their first year of Italian primary school; 22 of the bilingual children and 25 of the monolingual children were finishing their second year of Italian primary school. Thus, the children represented four groups: (a) 18 bilinguals with 1 year of Italian schooling, (b) 22 bilinguals with 2 years of Italian schooling, (c) 20 monolinguals with 1 year of Italian schooling, and (d) 25 monolinguals with 2 years of Italian schooling.

### Tasks

#### Background Measures

##### Parental background questionnaire

Children’s language background and exposure to both Italian and Sardinian was measured using an adapted version of the UBILEC, a comprehensive parental questionnaire measuring quantitative and qualitative aspects of language exposure (Unsworth, 2013a; Unsworth et al., 2014). The UBILEC questionnaire captures the amount of target language exposure over time in the past considering possible variation in early language development, such as language use during holiday and languages spoken in daycare or at school. To better quantify language competence in each language we looked at the information provided for each child by the cumulative language exposure index, which is part of UBILEC: this measured how much input was received from each parent and any other adults over time both at home and outside the home. The cumulative index is a detailed estimation of children’s language exposure over the years and a more accurate one compared to the traditional index of exposure that measures the differential amount of exposure between the languages (see Unsworth, 2013b for a detailed review). Children who scored lower than 3.3 on the UBILEC cumulative exposure index parameter for Italian were classified as bilingual. This was calculated as a median cut-off of the score reported for each child. Accordingly, 40 children were classified as bilingual and 45 children as monolingual. The bilingual children spoke Sardinian at home and in the community, and Italian at school. Given that Sardinian is the language commonly spoken in daily interactions in the Nuoro Province (Oppo, 2007), the monolingual children may also have been exposed to some Sardinian in the surrounding community, but Italian is the language spoken in their family as well as in day care or at school.

##### Raven’s Colored Progressive Matrix test

All children completed the Raven’s Colored Progressive Matrix (CPM) test of general intelligence (Raven et al., 1998) as an inclusion criterion to exclude any intellectual impairment. Children who performed within 2 SD of the age-corrected standardized score were included in the study.

##### Peabody Picture Vocabulary Test of receptive vocabulary (PPVT-4)

Recent discussions about the relative size of age-matched monolingual vs. bilingual children’s vocabulary (e.g., Bialystok, 2009; Bialystok et al., 2010) raise the possibility of differences in Italian language vocabulary between the monolingual and bilingual groups. The Peabody Picture Vocabulary Test of receptive vocabulary (PPVT-4, Stella et al., 2000) was therefore administered to all the children to establish their receptive Italian vocabulary knowledge. The test is incremental, and a basal score is established when the child makes six errors in eight consecutive responses. All children with a performance within 2 SD of the age normed transformed score were included in the study.

##### Digit span task

Several accounts suggest that areas of cognitive development (for example, executive function) are facilitated by short term memory (e.g., Gordon and Olson, 1998). Phonological memory was therefore assessed using a digit span test adapted from (Orsini et al., 1987; see Gathercole, 1998 for a review). For inclusion into the study, children had to show a digit span of ≥4 digits. No children were excluded.

##### Non-word repetition task

Non-word repetition has been shown to be a reliable index of verbal memory development and a clinical marker for detecting language impairment. A number of studies have reported that bilingual children are highly proficient in this task, sometimes showing an advantage over monolingual speakers (Tamburelli et al., 2015), but Guasti et al. (2013) found no differences between early second language learners and age-matched monolinguals Italian speakers. We therefore tested children on the non-word repetition task developed by Cornoldi et al. (2009) to exclude language impairment in both groups. To be included in the study, children had to achieve a non-word repetition score of at least 10 syllables. No children were excluded.

#### Test Measures

##### Receptive grammatical knowledge

Grammatical competence in Italian was measured using the COMPRENDO test (Cecchetto et al., 2012); a picture-matching task assessing sentence comprehension in Italian across syntactic structure types. The types of sentences included are all semantically reversible (with both nouns possibly acting as subject or object of the verb) and span structural complexity over seven conditions, shown in **Table 1**. As Section “Sardinian and Italian: A Brief Comparison,” Sardinian is both similar and different from Italian with respect to these structures. This is shown in **Table 1**.

**FIGURE 1:**
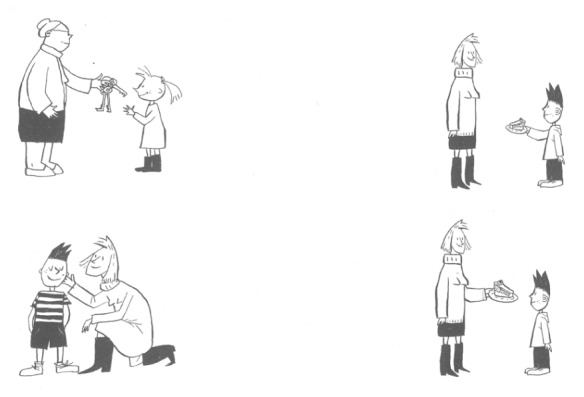
**COMPRENDO sample picture for dative target sentence “II bambino da la torta alia mamma” *(The boy gives the cake to the mother)***.

There were three items per condition with a total of 21 items per list, resulting in a 7 × 3 design. For each sentence, the child was asked to select one of four pictures (see example in **Figure 1**). The correct picture matched the sentence content: for the sentence “La mamma da la torta al bambino” (*The mum gives the cake to the boy)*, the picture showed a mother giving a cake to a young boy. In addition, there were three incorrect “distractor” pictures. The *reversal distractor* depicted the same actors in reversed roles (e.g., a boy giving a cake to his mother). The *verbal distractor* depicted the actors in the same thematic roles, but completing a different action (e.g., the mother caressing the boy). The *nominal distractor* kept the same action (e.g., giving), but replaced all the nouns (both the actors and the object; e.g., *The grandmother gives the keys to the girl*).

The task requires children to map the thematic roles (i.e., *Who is doing what to whom?*) in relation to the syntactic form of the sentence. This is a test of grammatical knowledge. However, general cognitive abilities such as executive control might be involved in this task, since competing interpretations have to be held in memory, and the incorrect ones must be inhibited.

##### Opposite world task

**FIGURE 2:**
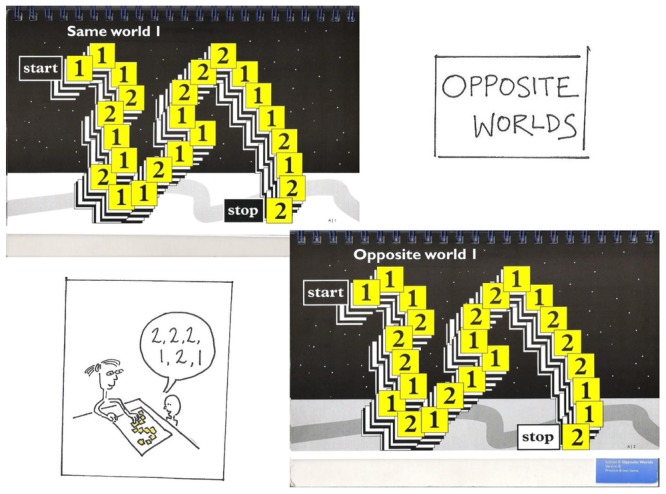
**Illustration of the ‘same’ and ‘opposite’ conditions in the Opposite World task (illustration courtesy of Ruth Cape)**.

This task is part of the Test for Everyday Attention for children (Manly et al., 1999, 2001) and is another common tool used to assess executive function in children. The children read a series of alternating numbers (e.g., 1, 2, 2, 1, 1, 2, 1, 2) aloud, in timed conditions. In the “same” condition, children read the numbers as they appear. In the “opposite” condition, children were asked to say the opposite of each digit (i.e., the previous sequence should be read as “2, 1, 1, 2, 2, 1, 2, 1”). An example is shown in **Figure 2**.

The variable of interest was the amount of time taken in the “opposite” condition, which requires inhibition of a prepotent verbal response: a faster response is taken to indicate an advantage in executive function.

##### Dimensional change card sort (DCCS)

A common measure of executive function in early childhood is the Dimensional Change Card Sort test (DCCS; Bialystok and Martin, 2004; see Zelazo, 2006 for the protocol adopted in this study). The standard version of this task requires children to sort a set of cards according to a particular dimension, such as color (e.g., “If it is blue it goes here, if it is red it goes there”); the children are subsequently asked to sort the same set of cards by according to a new dimension, such as shape (e.g., “If it is a rabbit it goes here, if it is a boat it goes there”). The test measures whether the child is able to switch from the first to the second dimension (marked as a 1), or instead, he/she keeps sorting the cards according to the first dimension (marked as 0). The variable of interest therefore is the number of correct responses.

### Procedure

Written informed consent was obtained from parents of all participating children in accordance with the Declaration of Helsinki. The study was approved by the Linguistics and English Language ethics committee at the University of Edinburgh.

Testing took place during school hours in a quiet room made available by the schools. Each child was involved in two experimental sessions, with a gap of one day between sessions. In the first session, which lasted approximately 30 min, four tasks were administered to children the following order: COMPRENDO, Opposite Worlds, DCCS, and Raven. In the second session, which lasted approximately 15 min, children performed the remaining background tests: PPVT, Digit Span, and non-word repetition tasks. All children performed all the tests in the same order. All tests were administered in Italian to both bilingual and monolingual children.

### Data Analyses

#### COMPRENDO

We used linear mixed effects (LME) models (e.g., Pinheiro and Bates, 2000) with logistic regression to estimate the likelihood of a correct response on a given trial. LME models with logistic regression have been demonstrated to handle categorical data (e.g., image selection) better than ANOVA (Jaeger, 2008). Mixed-effects modeling allows us to combine fixed effects (independent variables) with random effects terms sampled from a larger population, such as participant or item, thus capturing more of the random variance in a given data set (Baayen, 2008). All LME models were implemented in the lme4 package (Bates and Maechler, 2009) in R statistical software (R Development Core Team, 2011). All predictors were center prior to analysis, and coded using effects coding. This procedure helps to minimize collinearity (Baayen, 2008) and means that significance tests in the mixed-effects model correspond to tests for main effects and interactions in an ANOVA model (Cohen et al., 2003).

#### Opposite Worlds and DCCS Tasks

The opposite world task and DCCS produced a single statistic per child. Therefore, it was not possible to run LME models on these data, as random effects for participants or items were precluded. A standard linear model with age group, language group and their interactions as fixed effects was used instead.

## Results

### Background Measures

A summary of mean ages, cumulative exposure to Italian, and scores on background measures (RAVEN, PPVT-4, Digit span test, and non-word repetition) for the four age groups of participants is given in **Table 2**.

Linear models were used to test for significant differences between groups (language, age, and language by age) on the Raven CPM, PPVT-4, Digit span and non-word repetition background tests. Gaussian models were used for the Raven CPM, PPVT-4 and non-word repetition scores, and a Poisson model was used for the digit-span counts. Neither language (monolingual vs. bilingual), age (younger vs. older) or the interaction of language by age accounted for any significant difference in performance on the Raven CPM (Age-Language Group: est. -1.471, *SE* = 1.585, p. 0.36), PPVT-4 (Age-Language_Group: est. 4.78, *SE* = 5.57, p. 0.39, and Digit span tasks (Age-Language_Group: est. 0.12, *SE* = 0.20, p. 0.52). For the non-word repetition task, there was a main effect of age group, with the younger children making more errors than the older children (Age: est. 1.00, *SE* = 0.5, ^∗^*p* < 0.05), but no effect of language group or interaction between the two (Language_Group: est. 1.23, *SE* = 0.68, p. 0.07; Age-Language_Group: est -1.16, *SE* = 1.01, p. 0.25).

#### Test Measures

##### COMPRENDO

In the COMPRENDO task the children matched pictures to sentences of various levels of complexity. Recall that there were seven sentence types in total; active, passive, dative, coordinate, topicalised, subject relative, and object relative. We begin by analyzing all sentence types combined. We built an LME model using logistic regression. The dependent variable was the likelihood of a correct response on any given trial. The fixed effects were age group and language group, and their interactions. The model with maximal random effect structure failed to converge; this was a problem for all LME models in this section. We therefore removed the correlation parameter and the interaction term from the random slopes. This simplification resulted in a converged model and was used throughout these results unless otherwise specified.

**FIGURE 3:**
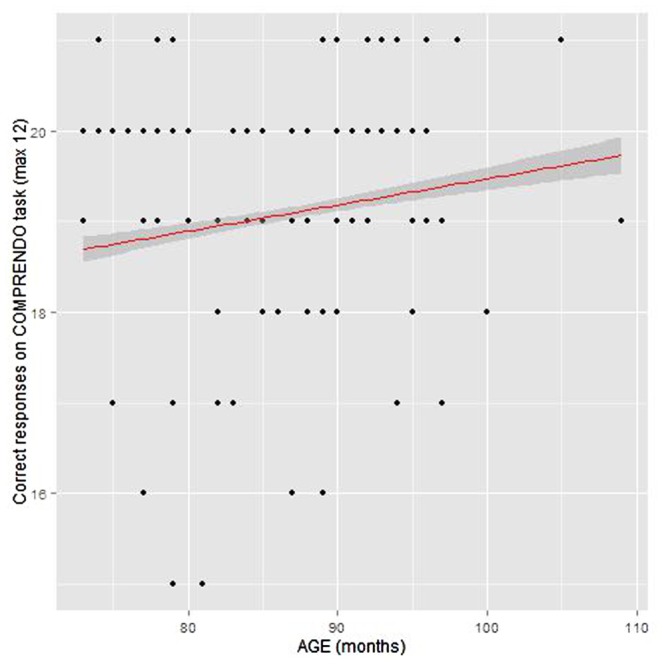
**Relationship between age and performance on COMPRENDO task**.

The average correct responses (of a maximum 21) across all groups was 19.10 (*SD* = 1.44; 91% correct). The model showed that children in their first year of schooling were significantly more likely to give a correct response (*M* = 18.79, *SD* = 1.54; 89% correct) than those in their second year of schooling (*M* = 19.34, *SD* = 1.31; 92% correct) **Figure 3**.

Bilingual children scored higher (*M* = 19.25, *SD* = 1.36; 91%) than monolingual children (*M* = 19.00, *SD* = 1.37; 90%), but this difference was not significant. The interaction between age group and language group was not significant. **Table 3** shows the model coefficients.

**Table 3:** Coefficients for linear mixed effects model in COMPRENDO: likelihood of correct response across all sentences combined ∼ Age group ^∗^ Language group.

	Estimate	*SE*	*p*
(Intercept)	2.88	0.30	<0.001^∗∗∗^
Age group	0.44	0.19	<0.05^∗^
Language group	-0.27	0.19	0.16
Age group: language group	-0.56	0.36	0.13

*^∗^*P* ≤ 0.05, ^∗∗∗^*P* ≤ 0.001*.

We then examined each type of sentence in turn. Performance by sentence type and by participant group is shown in**Table 4**. For active, passive, dative, coordinate, inflected, and subject relative sentences, there were no significant differences between age group or language group, and no significant interactions.

**Table 4:** COMPRENDO: performance by sentence type and participant group.

	Active (%)	Passive (%)	Dative (%)	Coordinate (%)	Inflected (%)	Subject relative (%)	Object relative (%)
Bilingual Y1	100	96	94	87	85	85	76
Bilingual Y2	100	97	97	97	88	88	92
Monolingual Y1	97	100	95	93	75	92	73
Monolingual Y2	100	99	97	92	84	84	80

**FIGURE 4:**
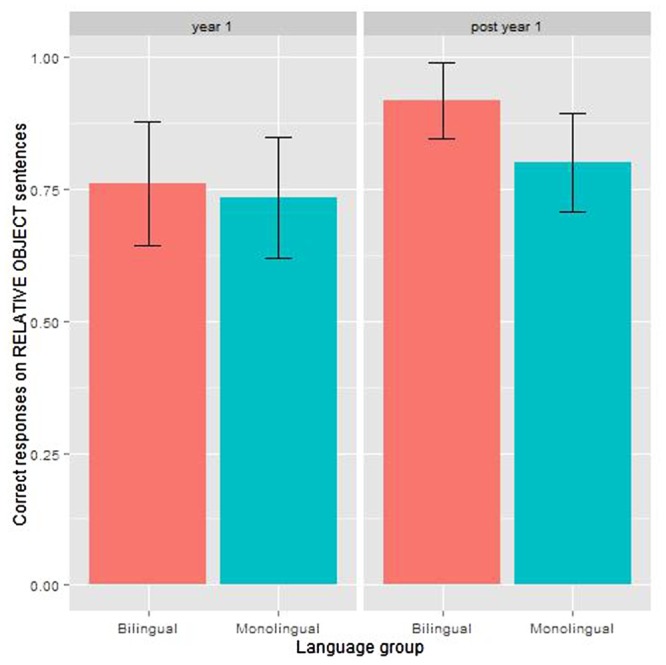
**Results for object relatives in the COMPRENDO test by language group and age group**.

For object relative sentences, the model showed a significant effect of age: older children answered correctly on 85% trials, compared with 75% for younger children (see **Figure 4**). The bilingual group was more likely to give a correct response (84% correct answers) than the monolingual group (77% correct answers), however, this was only marginally significant. There was no significant interaction between age and language group. **Table 5** shows the model coefficients.

**Table 5:** Coefficients for linear mixed effects model in COMPRENDO: likelihood of correct response to object relative sentences ∼ Age group ^∗^ Language group.

	Estimate	*SE*	*p*
(Intercept)	1.66	0.45	<0.001^∗∗∗^
Age group	0.84	0.36	<0.05^∗^
Language group	-0.67	0.39	0.08
Age group: language group	-0.92	0.72	0.20

*^∗^*P* ≤ 0.05, ^∗∗∗^*P* ≤ 0.001*.

#### Opposite World Task

A linear model was built in which the dependent variable was the amount of time taken in the “opposite” condition. The fixed effects were age group (first year of schooling or second year of schooling), and language group (monolingual or bilingual), and their interaction.

**FIGURE 5:**
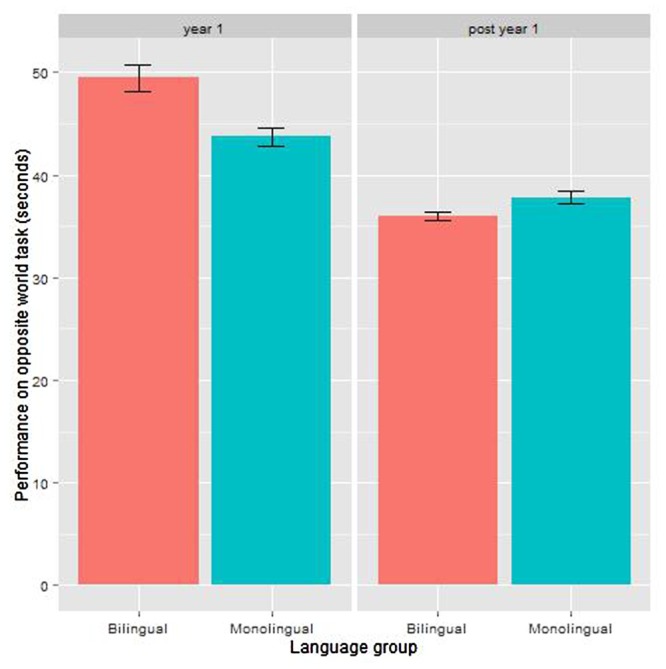
**Results in the Opposite World task by language group and age group**.

The average time across all age and language groups was 41.9 s (*SD* = 10.02). As expected, speed on this task decreased with age: the older the child, the faster they performed the task (see **Figure 5**). The linear model showed a significant effect of age: the older age group performed faster (*M* = 36.96, *SD* = 5.97) than the younger age group (*M* = 46.42, *SD* = 11.43). There was also a significant effect of language group, with bilingual children being slightly slower (*M* = 42.05, *SD* = 11.21) than monolingual children (*M* = 40.42, *SE* = 8.75); and this is mainly due to the younger bilingual children’s performance. The interaction between age group and language group is also significant: the bilingual children in their first year of schooling were 5.74 s slower on the task than their monolingual peers; bilingual children in their second year of schooling children were 1.8 s faster on the task than their monolingual peers. **Table 6** shows the model coefficients. See **Table 8** for mean and SD by group.

**Table 6:** Coefficients for linear model in opposite world: time taken in opposite world task ∼ Age group ^∗^ Language group.

	Estimate	*SE*	*T*	*p*
(Intercept)	41.18	0.20	202.03	<0.001^∗∗∗^
Age group	-9.45	0.41	-23.05	<0.001^∗∗∗^
Language group	-1.57	0.41	-3.85	<0.001^∗∗∗^
Age group:	7.54	0.82	9.19	<0.001^∗∗∗^
Language group				

*^∗∗∗^*P* ≤ 0.001*.

#### DCCS Task

A linear model was built in which the dependent variable was the number of correct answers from a maximum of 12. The fixed effects were age group (first year of schooling or second year of schooling), and language group (monolingual or bilingual), and their interaction.

**FIGURE 6:**
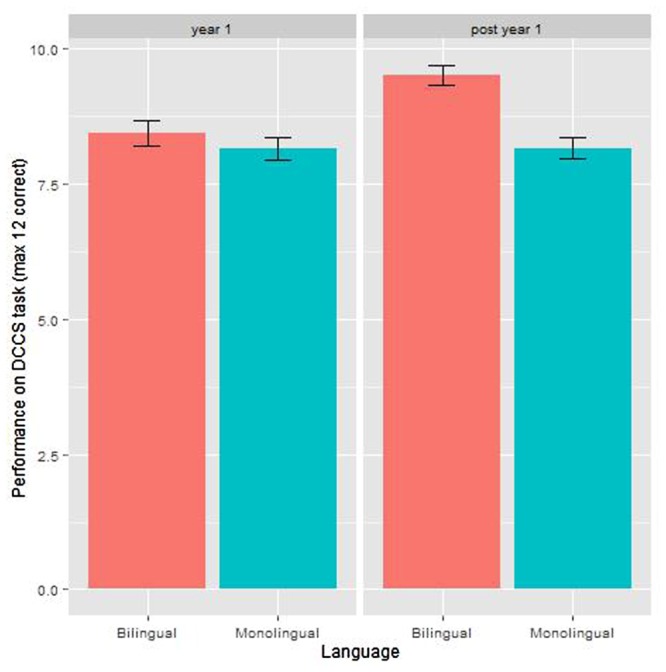
**Results of the DCCS by language group and age group**.

The average score across all groups was 8.57 (*SD* = 2.3). The linear model showed a significant effect of age, with children in their first year of schooling scoring lower (*M* = 8.29, *SD* = 2.27) than children in their second year of schooling (*M* = 8.79, *SD* = 2.33). There was also a significant effect of language group, with bilingual children scoring higher (*M* = 9.03, *SD* = 2.23) than monolingual children (*M* = 8.16, *SD* = 2.32); and this is mainly due to the older bilingual children’s performance (see **Figure 6**). The interaction between age group and language group is significant: the monolingual children’s score is more or less constant across years 1 and 2 of schooling, but the bilingual children in year 2 score higher than their bilingual peers in year 1. **Table 7** shows the model coefficients. See **Table 8** for mean and SD by group.

**Table 7:** Coefficients for linear model in DCCS: number of correct responses ∼ Age group ^∗^ Language group.

	Estimate	*SE*	*t*	*p*
(Intercept)	8.57	0.05	160.75	<0.001^∗∗∗^
Age group	0.50	0.11	4.684	<0.001^∗∗∗^
Language group	-0.87	0.11	-8.17	<0.001^∗∗∗^
Age group :	-1.05	0.21	-4.87	<0.001^∗∗∗^
Language group				

*^∗∗∗^*P* ≤ 0.001*.

**Table 8:** Mean performance for opposite world and DCCS tasks by participant group (SD in parentheses).

	Opposite world (seconds)	DCCS (correct responses/12)
Bilingual Y1	49.44 (12.51)	8.44 (2.31)
Bilingual Y2	36.00 (4.40)	9.50 (2.04)
Monolingual Y1	43.70 (9.61)	8.15 (2.22)
Monolingual Y2	37.80 (6.97)	8.16 (2.40)

## Discussion

The results of the study reported here can be summarized as follows:

(a) There are no significant differences between Sardinian–Italian bilingual children and monolingual Italian children in the control measures (i.e., Raven CPM, PPVT-4, Digit span, and non-word repetition).
(b) Overall, Sardinian–Italian bilingual children performed very similarly to monolingual Italian children in the COMPRENDO receptive grammatical test. All older children performed better than younger children, regardless of language group. There is a marginally significant difference in favor of bilinguals with respect to the comprehension of object relatives, which are the most complex of the seven syntactic structures tested: this difference is especially visible in older bilingual children.
(c) For the Opposite Worlds task, which is a test of executive functions requiring a verbal response, older children were overall faster than younger children. Younger bilingual children were slower than younger monolinguals whereas older bilinguals were faster than older monolinguals. This means that the score difference between younger and older children was wider for the bilingual group. Although the findings show an overall disadvantage for bilingual children, this is due to the large difference in performance between monolinguals and bilinguals in year 1, which is no longer present (and indeed, reversed – although not to the same extent) by year 2. The fact that this is a cross-sectional and not a longitudinal study invites caution in interpreting this difference as a steeper improvement in bilinguals. Furthermore, the verbal response required was in Italian, which may also have contributed to the monolingual advantage in younger children.
(d) For the DCCS, which is a task of executive function requiring a non-verbal response, there is improvement across the board from younger to older children. Younger bilinguals perform similarly to younger monolinguals. However, bilingual children provide more accurate responses in the older group.

These data reveal that bilingualism in Sardinian does not hinder development of linguistic competence in Italian, despite the fact that many of the bilingual children tested were dominant in Sardinian at the beginning of schooling. Bilingual children performed like monolinguals regardless of whether Sardinian and Italian are structurally similar or not. The trend toward bilingual advantages in comprehension of the object relative structure is more evident in older children. This may be regarded as further evidence that these advantages emerge gradually over time, as Bialystok et al. (2014) showed for children in immersion programs.

There is an alternative potential linguistic explanation for the trend toward a bilingual-monolingual difference in object relatives. In a study of adult learners of L2 Italian, Belletti and Guasti (2015) report that beginning L2 speakers are better than advanced L2 speakers, and often show ceiling performance in the production of object relatives. A very low percentage of passive object relatives were attested in beginner L2 speakers (22%) compared to a much higher production of passive object relatives in advanced L2 speakers (60%). In contrast, beginning L2 speakers produced 77% of correct object relatives compared to just 15% in the advanced group, approaching the performance of native Italian speakers. The low attested productions of passive object relatives in low proficiency Italian L2 speakers seems to mirror the finding of the present study that Sardinian–Italian bilingual children are marginally better at comprehending object relatives. Belletti and Guasti (2015) suggest that avoidance strategies are not available at early stages of acquisition in L2 speakers possibly due to a still imperfect command of morphosyntactic features of the language. It is unclear how avoidance strategies would affect comprehension. Notice, however, that ‘imperfect command’ here is not necessarily to be understood as lack of relevant knowledge, but possibly as slower access to alternative structures that may compete with object relatives. It is also possible that bilingual children may have sufficient inhibitory control to exclude the alternative structures. These differences cannot be directly tested in this study, and further research is necessary to explore these alternative accounts.

The analysis of the cognitive test results points to a global improvement from younger to older children, and to an overall advantage for older bilingual children. The Opposite World test and the DCCS both test aspects of executive functions, such as the ability to inhibit an inappropriate response and switch between conditions. Only the Opposite World test, however, requires an overt verbal response (in Italian). It is in this test that younger bilingual children (whose home language is Sardinian, rather than Italian) have an initial disadvantage compared to monolinguals. In the DCCS, on the other hand, bilinguals and monolinguals are the same in the younger group. A plausible interpretation of this disparity between the tests may be related to the fact that the bilinguals in the first year of primary school had experienced comparably fewer opportunities to use Italian productively. As was the case for the COMPRENDO test, advantages in cognitive function may emerge gradually with time and more exposure to both languages. In any case, bilingualism involving a regional minority language may come with some of the same beneficial effects as bilingualism in other languages.

## Conclusion

This study involved 85 children from the Nuoro province of central Sardinia, of whom 45 were monolingual in Italian and 40 were bilingual in Sardinian and Italian. All children were comparable with respect to vocabulary knowledge, phonological memory, typical language development, and general intelligence. The children performed in a test of Italian receptive competence and in two standardized tests of executive functions. In most cases the performance of bilingual children was not different from monolinguals.

This study has limitations. The most obvious ones are the limited size of the sample, the cross-sectional design, and the narrow range of abilities tested. Future research will explore the relationship between comprehension and production abilities in the Italian of Sardinian–Italian children, as well as the correlations between language abilities and cognitive abilities. The full range of abilities should be studied over a longer period of time, in both longitudinal and cross-sectional studies, to establish the developmental trajectories of both linguistic and general cognitive skills, and of the effects on each other. Despite these limitations, however, the results of this study are inconsistent with the common perception that bilingualism with Sardinian is a cognitive burden and compromises performance in Italian.

## Author Contributions

MG: conception and design of the work; analysis and interpretation of data for the work; drafting the work and revising it critically for important intellectual content; final approval of the version to be published; agreement to be accountable for all aspects of the work in ensuring that questions related to the accuracy or integrity of any part of the work are appropriately investigated and resolved.

MB: analysis and interpretation of data for the work; drafting the work and revising it critically for important intellectual content; final approval of the version to be published; agreement to be accountable for all aspects of the work in ensuring that questions related to the accuracy or integrity of any part of the work are appropriately investigated and resolved.

AS: conception and design of the work; interpretation of data for the work; drafting the work and revising it critically for important intellectual content; final approval of the version to be published; agreement to be accountable for all aspects of the work in ensuring that questions related to the accuracy or integrity of any part of the work are appropriately investigated and resolved.

## Conflict of Interest Statement

The authors declare that the research was conducted in the absence of any commercial or financial relationships that could be construed as a potential conflict of interest. The reviewer and handling Editor declared their shared affiliation, and the handling Editor states that the process nevertheless met the standards of a fair and objective review.
